# Case report: Eculizumab plus obinutuzumab induction in a deceased donor kidney transplant recipient with DEAP-HUS

**DOI:** 10.3389/fimmu.2022.1073808

**Published:** 2022-12-14

**Authors:** Evaldo Favi, Paolo Molinari, Carlo Alfieri, Giuseppe Castellano, Mariano Ferraresso, Donata Cresseri

**Affiliations:** ^1^ General Surgery and Kidney Transplantation, Fondazione IRCCS Ca’ Granda Ospedale Maggiore Policlinico, Milan, Italy; ^2^ Department of Clinical Sciences and Community Health, Università degli Studi di Milano, Milan, Italy; ^3^ Nephrology, Dialysis, and Transplantation, Fondazione IRCCS Ca’ Granda Ospedale Maggiore Policlinico, Milan, Italy

**Keywords:** kidney transplant, atypical hemolytic uremic syndrome, anti-complement factor H antibody, CFHR1/CFHR3 gene mutation, DEAP-HUS, eculizumab, obinutuzumab, case report

## Abstract

The wide-spread use of the anti-complement component 5 monoclonal antibody (moAb) eculizumab has greatly reduced the incidence of relapsing atypical hemolytic uremic syndrome (aHUS) after kidney transplantation (KT). However, the optimal management of aHUS transplant candidates with anti-Complement Factor H (CFH) antibodies remains debated. In these patients, the benefits of chronic eculizumab administration should be weighed against the risk of fatal infections, repeated hospital admissions, and excessive costs. We report the case of a 45-year-old female patient with *CFHR1/CFHR3* homozygous deletion-associated aHUS who underwent deceased-donor KT despite persistently elevated anti-CFH antibody titers. As induction and aHUS prophylaxis, she received a combination of eculizumab and obinutuzumab, a humanized type 2 anti-CD20 moAb. The post-operative course was uneventful. After 1-year of follow-up, she is doing well with excellent allograft function, undetectable anti-CFH antibodies, sustained B-cell depletion, and no signs of aHUS activity. A brief review summarizing current literature on the topic is also included. Although anecdotal, our experience suggests that peri-operative obinutuzumab administration can block anti-CFH antibodies production safely and effectively, thus ensuring long-lasting protection from post-transplant aHUS relapse, at a reasonable cost. For the first time, we have demonstrated *in vivo* that obinutuzumab B-cell depleting properties are not significantly affected by eculizumab-induced complement inhibition.

## 1 Introduction

Atypical hemolytic uremic syndrome (aHUS) is a rare thrombotic microangiopathy (TMA) characterized by uncontrolled activation of the alternative pathway of the complement system, with an acute-phase mortality rate ranging from 2% to 10%, and progression to end-stage renal disease (ESRD) in about half of the patients (1–3). Extra-renal manifestations (mainly, hepatic and neurological) are frequent and can be observed in up to 20% of the cases. The disease, often triggered by infections, pregnancy, delivery, malignancy, medications, surgery, or organ transplantation, is mostly due to mutations in genes encoding complement regulatory proteins such as Complement Factor H (CFH), Complement Factor I (CFI), Membrane Cofactor Protein (MCP), Complement Factor B (CFB), or Complement Component 3 (C3) (4–6). Anti-CFH antibodies have also been recognized as a possible pathogenic factor, being detected in approximately 10% of the subjects with aHUS, especially children and teenagers (7–9).

Although we have witnessed tremendous advancements in the management of aHUS over the last decade, disease variants due to CFH functional deficiency still represent a major challenge (10). Among the others, anti-CFH antibody-related aHUS deserves special consideration as it is associated with excessive morbidity and mortality (10–13). Anti-CFH antibodies bind to the C-terminal domain of CFH, thus determining CFH dysfunction and complement dysregulation (14). Anti-CFH antibody-related aHUS is a multisystemic disease with frequent gastrointestinal involvement (15), a strong association with cardiovascular events (16–18), and a characteristic relapsing-remitting course. Overall mortality surpasses 10%, and recent data show recurrence or progression to ESRD in more than 50% and 30% of the patients, respectively (17). In clinical practice, anti-CFH antibody levels are used as a surrogate biomarker of disease activity, with sudden and sharp rises in titers often preceding recrudescence or relapse (17, 19, 20). This specific aHUS population can be sorted in two groups: patients without any genetic background promoting anti-CFH antibodies production (at lower risk of recurrence after anti-CFH antibodies reduction or disappearance), or those with a genetically-driven form of anti-CFH antibody-related aHUS (at higher risk of relapse and requiring extreme caution in terms of diagnosis and management) (9, 21). The latter subset of patients exhibits a unique combination of genetic and acquired predisposing factors for post-transplant aHUS recurrence. The acquired factor is the presence of circulating anti-CFH antibodies whereas the genetic factor is represented by a homozygous deletion of an 84-kb fragment located on chromosome 1, encompassing the *CFH-Related protein 1* (*CFHR1*) and *CFH-Related protein 3* (*CFHR3*) encoding genes (so called, DEficiency of CFHR plasma proteins and Autoantibody Positive form of Hemolytic Uremic Syndrome, DEAP-HUS). How the lack (or reduction) of circulating CFHR1 and CFHR3 promotes the development of anti-CFH antibodies is unclear. Nonetheless, it is plausible that patients with a genetic (and unmodifiable) background might be more prone to disease relapse, therefore requiring life-long and targeted interventions to block or decrease anti-CFH antibodies production (9, 16, 19, 22–24).

To date, there is no consensus regarding the management of DEAP-HUS. Plasma exchange (PE) is widely used for rapid antibodies removal and functional CFH replenishment. However, after PE withdrawal, most patients experience a progressive increase in anti-CFH antibody levels and disease recurrence. Long-term PE feasibility and efficacy are further limited by the frequent occurrence of severe adverse reactions and complications (25). To reduce rebound antibody production, chronic immunosuppression with different combinations of steroids, cyclophosphamide, mycophenolate mofetil (MMF), cyclosporine, or tacrolimus have been proposed, with mixed results. Intravenous human polyclonal immunoglobulins and the type 1 anti-CD20 monoclonal antibody (moAb) rituximab have been administered in some series, aiming to obtain immunomodulation and sustained B-cell depletion (8, 17, 20, 26–34). The chronic use of the anti-complement component 5 (C5) moAb eculizumab is mostly restricted to patients with persistently elevated circulating anti-CFH antibodies and additional aHUS-inducing genetic abnormalities (23, 32).

Overall, experience in kidney transplant (KT) setting is scarce. The current practice favors a personalized approach. Patients with undetectable anti-CFH antibody levels at the time of transplant and no additional complement system abnormalities are at low risk of recurrence, and do not generally receive peri-operative aHUS prophylaxis. Low-to-high anti-CFH antibody titers without concomitant complement system abnormalities entail an intermediate risk of relapse. Therefore, in these recipients, pre-operative PE with a single shot of eculizumab are advocated. In individuals with persistently elevated anti-CFH antibody levels and aHUS-associated genetic mutations, the risk of relapse is high, and repeated pre- and post-transplant PE sessions with chronic eculizumab administration are recommended (35, 36).

For the first time, we report the prophylactic use of eculizumab in association with the type 2 anti-CD20 moAb obinutuzumab in a deceased-donor KT recipient with *CFHR1/CFHR3* homozygous deletion-associated aHUS and circulating anti-CFH antibody.

## 2 Case description

A 45-year-old female patient with ESRD due to DEAP-HUS underwent pre-emptive KT on May 2021. Her comorbidities included systemic hypertension with moderate organ damage (hypertensive retinopathy and left ventricular hypertrophy) and secondary hyperparathyroidism. The onset of aHUS was supposedly recorded at the age of seven (1975), when she had been admitted to her hometown hospital after several episodes of loss of consciousness associated with elevated blood pressure and seizures. Clinical examination, laboratory tests, and renal pathology showed signs of TMA, with severe acute kidney injury and histological features of necrotizing glomerulonephritis. The course of the episode was self-limiting, and the patient received high-intensity supportive care, blood transfusions, and temporary renal replacement therapy. For many years, the disease remained clinically quiescent. Nevertheless, at the age of 27 (1994), after a complicated pregnancy, she started experiencing multiple relapses eventually leading to progressive kidney failure, despite repeated hospital admissions and PE sessions. In 2013, the patient was referred to a local low-clearance clinic where no aHUS-targeted therapy was administered. For seven years, no further episodes of disease recurrence were recorded, but her renal function slowly deteriorated. In 2020, she attended our pre-transplant assessment clinic. Screening laboratory tests revealed decreased C3 plasma levels (66 mg/dL; normal range, 90–180 mg/dL). For this reason, the patient was evaluated at the affiliated Center for the Study and Therapy of aHUS. Following genetic studies and specific investigations, a definitive diagnosis of aHUS due to *CFHR1/CFHR3* homozygous deletion and anti-CFH antibodies was made. Her father (87 y/o), mother (81 y/o), brother (59 y/o), and sister (47 y/o) refused genetic evaluation; their past medical history was unremarkable. Her son (28 y/o) showed *CFHR1/CFHR3* heterozygous deletion without any clinical or laboratory signs of aHUS. During the time spent on the transplant waiting list, anti-CFH antibodies remained persistently elevated, with lowered C3 plasma levels. Although there were features of possible sub-clinical aHUS activity, no PE or complement-targeted therapy was administered, because the loss of renal function was deemed irreversible, and the risk-benefit ratio was in favor of a conservative approach until transplant.

The kidney donor was a 40-year-old male subject who had died of sudden cardiac arrest after few days spent in intensive care unit. Ante mortem serum creatinine concentration (SCr) was normal. The kidney was medium-sized, had one artery, one vein, and one ureter. The donor and the recipient were blood group compatible and had a 4 HLA antigen mismatch. Maximal and pre-operative panel reactivity antibody (PRA) tests were 5% and 0%, respectively. Flow and direct micro-cytotoxicity cross-match were both negative, but two non-donor-specific anti-HLA antibodies with a mean fluorescence intensity (MFI) above 3000 were detected (likely, as a result of previous immunization following repeated transfusions). At the time of surgery, C3 levels were low (70 mg/dL) and circulating anti-CFH antibodies were elevated (13 AU/mL; normal range, 0–5 AU/mL). As required for transplant eligibility, there were no signs of clinically active aHUS or hemolytic anemia (normal LDH, haptoglobin, and platelet). The graft was extra-peritoneally positioned in the right iliac fossa as per standard practice. The entire procedure took 180 minutes, with neglectable intra-operative blood loss.

**Figure 1:**
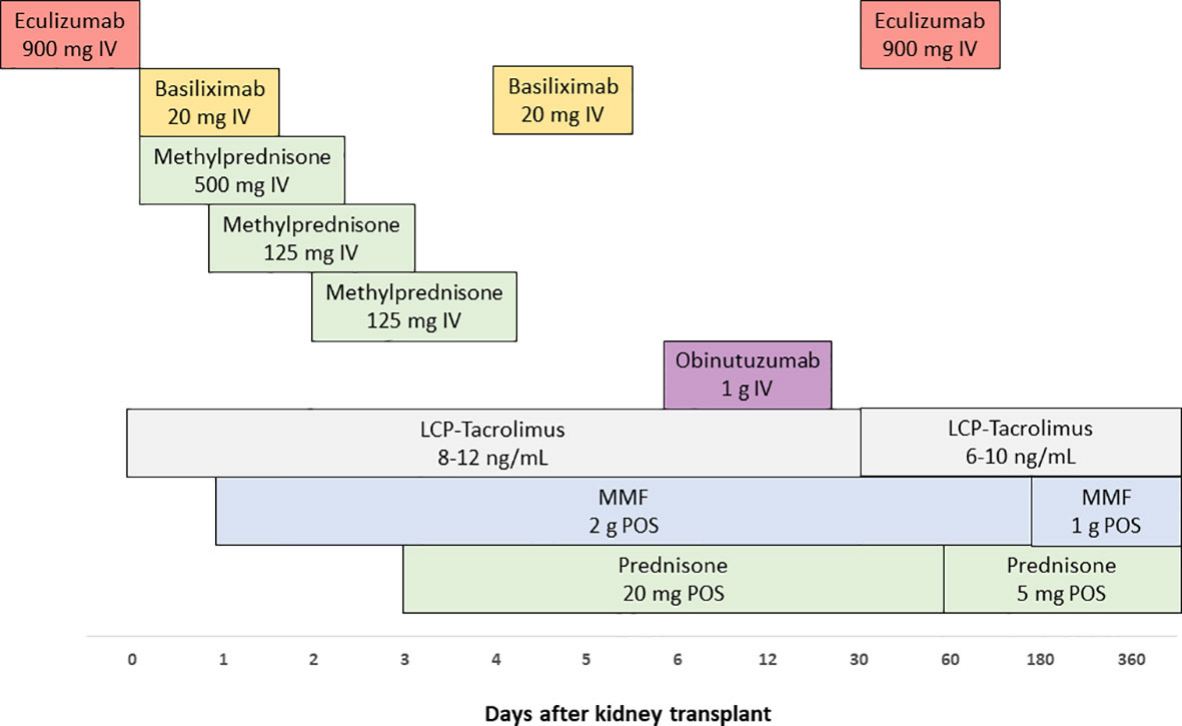
Detailed timeline of kidney transplant-related interventions.

**Figure 2:**
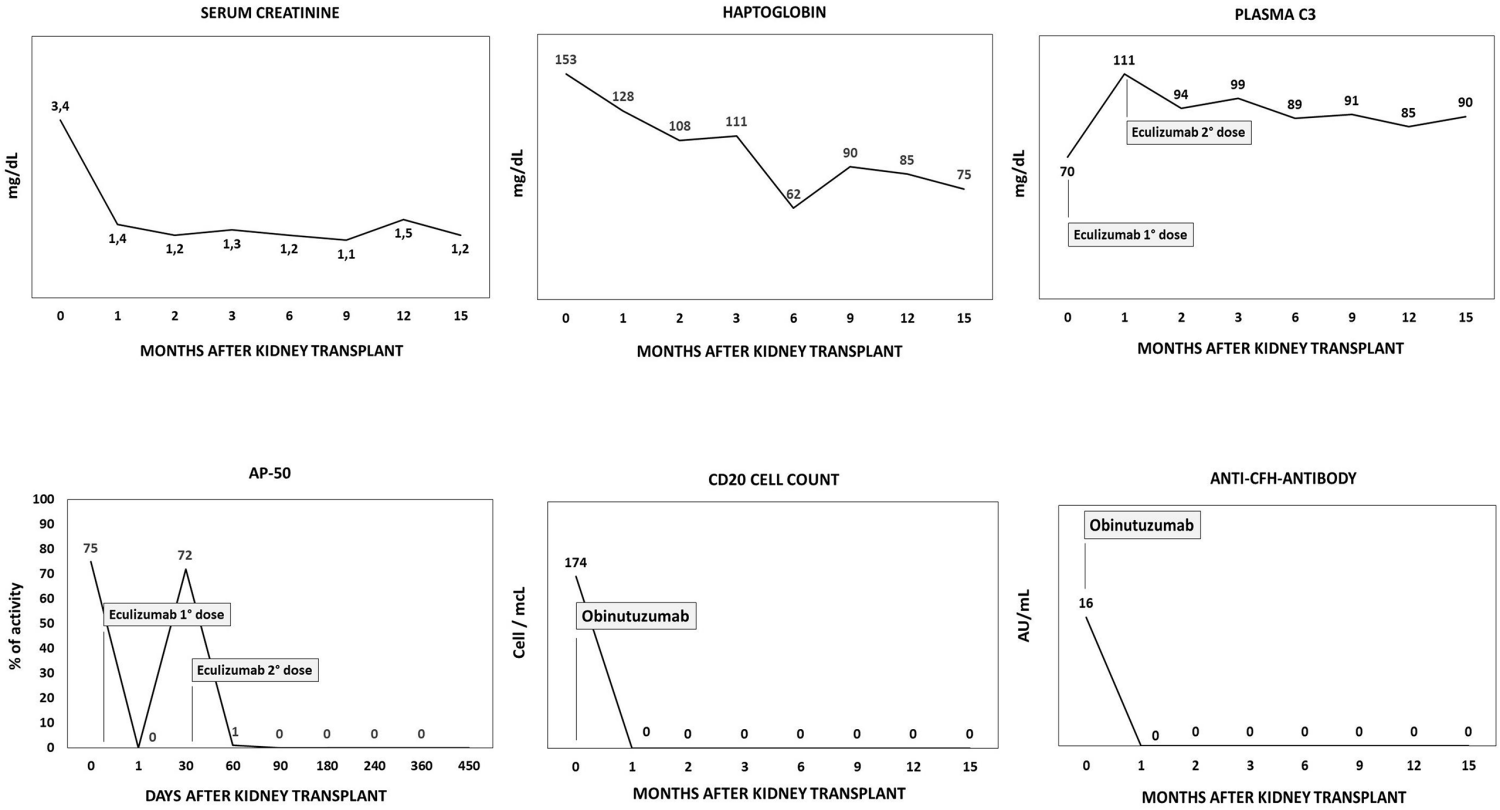
Main peri-transplant and post-transplant DEAP-HUS-related parameters.

Considering the past medical history, the diagnosis of aHUS due to *CFHR1/CFHR3* heterozygous deletion and circulating anti-CFH antibodies, the pre-operative C3 depletion, and the pre-operative anti-CFH antibodies positivity, the patient was considered at high risk of post-KT recurrence. Therefore, following multidisciplinary discussion, she received peri-operative complement inhibition and B-cell depletion. More in details, as induction, the recipient was given eculizumab (Soliris^®^, Alexion Pharmaceutical, Boston, MA) 900 mg IV one hour before surgery, basiliximab (Simulect^®^, Novartis, Basel, Switzerland) 20 mg IV prior reperfusion and on post-operative day 4, methylprednisolone (500 mg IV intra-operatively, 125 mg IV on day 1, and 125 mg IV on day 2), and obinutuzumab (Gazyvaro^®^, Hoffman-La Roche, Basel, Switzerland) 1000 mg IV on post-transplant day 6. As maintenance immunosuppression, we used oral LCP-tacrolimus (Envarsus^®^, Chiesi, Parma, Italy), MMF (Myfenax^®^, Teva, Petach Tikva, Israel), and prednisone. LCP-tacrolimus was started on admission and the dose was adjusted to achieve a trough level of 8-12 ng/mL during the first month, and 6-10 ng/mL thereafter. MMF was administered starting on post-operative day 1 using 1000 mg twice daily. From day 3, the patient also received prednisone 20 mg/day, progressively tapered to 5 mg/day after two months of follow-up. The early post-operative course was uneventful, and no delayed graft function (DGF), rejection, aHUS relapse, surgical, or infectious complications were recorded. After 12 days of hospitalization, she was discharged with a SCr of 1 mg/dL. Obinutuzumab infusion was not associated with any drug-related adverse reactions. Although the complement system had been effectively inhibited by eculizumab (AP50 and CH50 less than 5%), a single administration of obinutuzumab determined prompt and complete B-cell depletion (undetectable CD20 and CD19 cell counts within 24 hours of infusion) and persistent anti-CFH antibody disappearance. On post-transplant day 30, due to a transient rise in AP50, a second dose (1200 mg IV) of eculizumab was administered for prophylactic purposes. After one year of follow-up, the recipient is doing very well, with excellent allograft function, no detectable anti-CFH antibody, normal complement activity, no laboratory signs of hemolytic anemia, and sustained B-cell depletion. Excluding few episodes of asymptomatic Cytomegalovirus viraemia, easily managed with oral valganciclovir and temporary MMF minimization, she did not experience any significant complications (**Figures 1**, **2**). Her perspective regarding the overall experience and the specific treatments received was very positive.

## 3 Discussion

**Figure 3:**
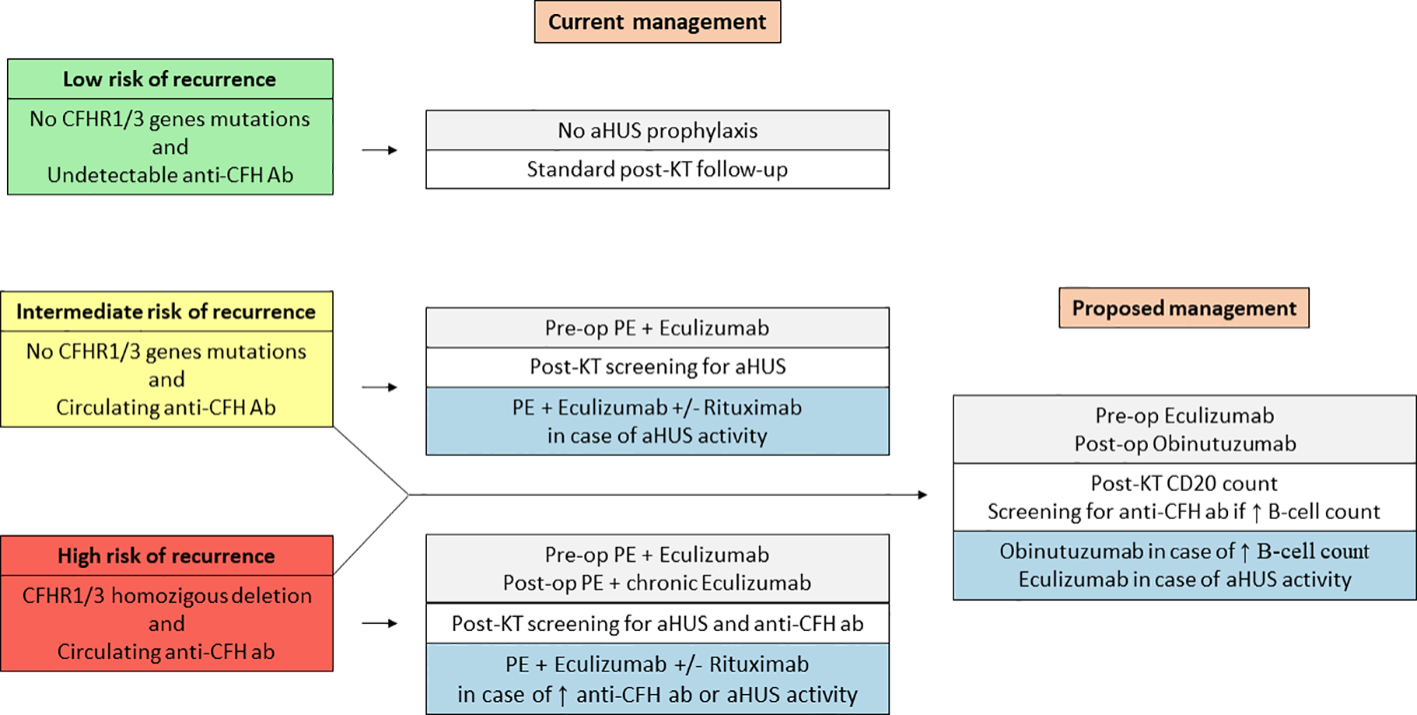
Current and proposed management of kidney transplant recipients with DEAP-HUS.

The introduction of eculizumab into clinical practice has drastically changed the management of aHUS, leading to improved patient survival and reduced morbidity (37). However, the optimal treatment of patients with DEAP-HUS remains debated. Due to the rarity of the disease and the heterogeneity of the population involved, there is a lack of evidence supporting a specific prophylactic approach over the other ones (8, 32, 38). Data on KT recipients with anti-CFH antibody-associated aHUS are limited to few cases (**Table 1**). Current literature suggests that KT candidates with elevated anti-CFH antibody titers carrying a homozygous deletion of *CFHR1/CFHR3* encoding genes require long-term prophylaxis to prevent allograft loss due to aHUS recurrence (**Figure 3**). The ideal prophylactic protocol should ensure prompt anti-CFH antibody removal, effective complement inhibition, and long-lasting blockage of anti-CFH antibody production. Antibody removal is generally achieved using an aphaeretic technique performed before the transplant procedure and during the post-transplant course, in case of antibody persistence or rebound (20, 25). For sustained complement inhibition, life-long eculizumab represents the preferred option (40–42) whereas to achieve anti-CFH antibody suppression, chronic administration of anti-proliferative compounds or repeated use of B-cell depleting agents can be considered (14, 33, 41, 43). To the best of our knowledge, this is the first report describing the combined use of eculizumab and obinutuzumab in DEAP-HUS. Also, our contribution represents a unique source of information on obinutuzumab-based induction in deceased-donor KT, as well as the demonstration *in vivo* of effective obinutuzumab-induced B-cell depletion in the context of iatrogenic complement inhibition.

**Table 1:** Previously published reports of kidney transplantation in patients with DEAP-HUS.

Ref.	Age at KT/KT#	DEAP-HUS mutations	DEAP-HUS prophylaxis	KT type/Immunosuppression	Follow-up/Outcomes
20	NA/ 3	NA	IVIG + RTX	LD (n=2); DD (n=1)/ NA	6–15mo FU/ 3 KTs: no recurrence
23	12y/ 1	- hom *CFHR1/3* deletion - *C3* mutation	NA	NA/ NA	3y FU/ no recurrence
	11y/ 1	- no hom *CFHR1/3* deletion	NA	NA/ NA	2y FU/ no recurrence
	12y/ 1	- hom *CFHR1/3* deletion - *CFH* mutation	NA	NA/ NA	6y FU/ no recurrence
17	NA/ 1	NA	- PE - RTX	NA/ NA	8mo FU/ no recurrence
	NA/ 1	NA	NA	NA/ NA	NA/ recurrence
16	6y/ 1	- hom *CFHR1/*3 deletion	NA	NA/ NA	16y FU/ no recurrence
	4y/ 1	- no *CFHR1/3* mutations	NA	NA/ NA	8mo FU/ no recurrence
27	12y/ 1	- no *CFH* mutation	- PE - RTX - azathioprine - prednisone	NA/ - basiliximab - tacrolimus - MMF - prednisone	2y FU/ no recurrence
28	7y/ 5	hom *CFHR1/3* deletion	- PE	NA/ NA	0-13y FU/ 1 KT: recurrence with graft loss 1 KT: TMA with graft loss 2 KTs: IF/TA with graft loss 1 KT: no recurrence
35	NA/ 1	hom *CFHR1/3* deletion	- PE	NA/ NA	5y FU/ recurrence treated with eculizumab
33	11y/ 1	hom *CFHR1/3* deletion *CFH* variant G2850T	- basiliximab - RTX	DD - basiliximab - MMF - prednisone	6y FU/ no recurrence
39	NA/ 1	NA	NA	NA/ NA	NA/ recurrence with graft loss
8	14y/ 1	hom *CFHR1/3* deletion *CFI* polymorphism	- PE - ATG - IVIG	LD/ - cyclosporin - tacrolimus - MMF	3y FU/ no recurrence (anti-CFH Ab +)

KT, kidney transplant; DEAP-HUS, DEficiency of CFHR plasma proteins and Autoantibody Positive form of Hemolytic Uremic Syndrome; NA, not available; IVIG, intravenous immunoglobulin; RTX, rituximab; LD, living donor; DD, deceased donor; FU, follow-up; mo, months; y, years; hom, homozygous; CFHR, complement factor H receptor; CFH, complement factor H; PE, plasma exchange; MMF, mycophenolate mofetil; TMA, thrombotic microangiopathy; CFI, complement factor I; ATG, anti-thymocyte globulin; Ab, antibody.

Having a previous history of multiple relapses, as well as *CFHR1/CFHR3* homozygous deletion and circulating anti-CFH antibodies, our transplant candidate was at high risk of relapse. In line with current trends, we used pre-operative eculizumab for prompt complement inhibition in the very early post-transplant phase, when the risk of recurrence was highest (41). However, rather than using pre-and post-operative PE for anti-CFH antibody removal, we opted for antibody production blockage through the administration of an anti-CD20 moAb. The rationale behind this strategy recognizes several considerations. First, we aimed to avoid pre-transplant PE as it would have determined a significant elongation of cold ischemia time, increasing the risk of DGF and acute rejection. Also, PE could have been associated with adverse reactions, leading to further delay or temporary inability to proceed with transplantation (44–46). Secondly, our goal was to prevent long-term aHUS recurrence without repeated PE or chronic eculizumab administration, thus reducing patient’s discomfort, drug-related adverse events, and costs (36, 43). In this regard, sustained B-cell depletion (and subsequent antibody production inhibition) appeared as the most suitable option.

Obinutuzumab is a type 2 humanized anti-CD20 moAb primarily developed to treat B-cell malignancies (47). Two very recent studies in highly sensitized KT candidates have shown more effective B-cell depletion and improved transplant rates compared to rituximab, with better tolerability and reduced incidence of severe adverse reactions (48, 49). Moreover, there is evidence *in vitro* that, unlike rituximab (50), obinutuzumab does not necessarily require complement activation for B-cell depletion (51), alternatively inducing B-cell death *via* a complement-independent pathway (52). Further characteristics in favor of obinutuzumab are increased binding affinity to FcRIIIa on target B-cells (with enhanced stimulation of antibody-dependent cell cytotoxicity and maintained function in the presence of an excess of polyclonal IgG), superior pro-apoptotic properties (activation of a non-caspase dependent cell death), and reduced incidence of drug resistance due to the formation of anti-obinutuzumab antibodies (48, 49, 51).

In our case, obinutuzumab ensured complete and sustained B-cell depletion, as well as prolonged anti-CFH antibody blockage, despite eculizumab-induced complement inhibition, thus demonstrating *in vivo* what previously observed in experimental models. Remarkably, obinutuzumab administration was not associated with any infusion-related reactions or severe drug-related complications (48, 49, 51). The strategy herein proposed was also extremely cost-effective as repeated PE sessions or eculizumab infusions would have been more expensive than a single dose of obinutuzumab (51). Guiding post-transplant DEAP-HUS prophylaxis with serial evaluations of CD20 count rather than AP50, CH50, C4, and C3 determinations represents an opportunity to further reduce costs, and could be easily performed as an outpatient in local facilities, outside academic or high-volume hospitals. Indeed, we believe that most DEAP-HUS KT patients can be safely treated with a tailored approach, possibly using peri-operative complement inhibition and prolonged B-cell suppression, thus avoiding the risks, costs, and discomfort associated with repeated PE or life-long eculizumab administration. During the post-transplant follow-up, additional obinutuzumab doses may be considered in case of B-cell repopulation with concomitant anti-CFH antibodies reappearance. Accordingly, extra eculizumab administrations should be proposed only in case of clinically active TMA. Moreover, in sensitized recipient, obinutuzumab induction could reduce the risk of antibody-mediated rejection, with improved long-term allograft function and survival.

Despite the encouraging results and the reproducibility of the induction scheme herein described, we recognize the anecdotal value of the present report as much as the need for validation with properly designed trials. Also, it would have been interesting to assess circulating CFHR1 and CFHR3 levels during the diagnostic work up. Further studies are warranted to confirm the efficacy of obinutuzumab for anti-CFH antibody blockage, possibly defining the risks and benefits associated with prolonged B-cell depletion compared to chronic complement inhibition. The feasibility of using high-dose antiproliferative agents instead of repeated anti-CD20 moAb in patients with stable allograft function and sustained anti-CFH antibody suppression, should also be explored.

## Data availability statement

The raw data supporting the conclusions of this article will be made available by the authors, without undue reservation.

## Ethics statement

Ethical review and approval were not specifically required for the present case report in accordance with the local legislation and institutional requirements. The subject involved has formally consented to enlistment in the kidney transplant waiting list, kidney transplantation, transplant-related treatments (including label and off-label use), and follow-up investigations. A dedicated consent for data collection and analysis has been obtained from all the patients referred and admitted to our hospital during the COVID-19 pandemic. Furthermore, written informed consent was obtained from the individual for the publication of any potentially identifiable images or data included in this article.

## Author contributions

EF and DC: rationale of the study, original draft of the manuscript, final revision; PM and CA: data collection and interpretation, literature review, editing; GC and MF: supervision, logistics, final revision. All authors contributed to the article and approved the submitted version.
